# The concept of immune surveillance against tumors: The first theories

**DOI:** 10.18632/oncotarget.12739

**Published:** 2016-10-18

**Authors:** Domenico Ribatti

**Affiliations:** ^1^ Department of Basic Medical Sciences, Neurosciences and Sensory Organs, University of Bari Medical School, Bari, Italy; ^2^ National Cancer Institute Giovanni Paolo II, Bari, Italy

**Keywords:** Antigen, immune surveillance, history of medicine, T cell, tumor

## Abstract

The immune system plays a major role in the surveillance against tumors. To avoid attack from the immune system, tumor cells develop different strategies to escape immune surveillance. Evidence of immune surveillance comes from both animal models and clinical observations. Mice with a wide variety of immunodeficiencies have a high rate of tumor incidence and are more susceptible to transplanted or chemical carcinogen-induced tumors. Immunosuppressed patients have a high incidence of tumors. However, many patients develop cancer even in the presence of an apparently normal immune system. This indicates that tumor cells are able to escape immune surveillance. The aim of this review article is to summarize the literature concerning the development of the theory of immune surveillance against tumors; to discuss the evidence for and against this theory, and to discuss the concept of immunoediting. Finally, the current approaches in anti-tumor immunotherapy will be analyzed.

## INTRODUCTION

**Figure 1:**
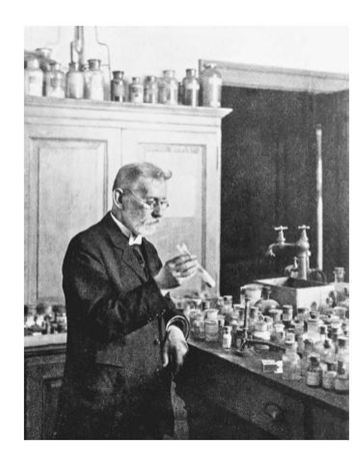
A portrait of Paul Ehrlich

In 1909, Paul Ehrlich (Figure 1) formulated the hypothesis that host defense may prevent neoplastic cells from developing into tumors [1]. He stated that: “in the enormously complicated course of fetal and post-fetal development, aberrant cells become unusually common. Fortunately, in the majority of people, they remain completely latent thanks to the organism's positive mechanisms.” [1]. This hypothesis was not proven experimentally at the time due to the inadequacy of experimental tools and knowledge.

**Figure 2:**
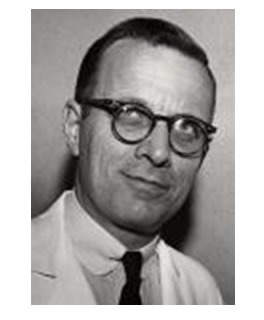
A portrait of Lewis Thomas

Later, some biologists suggest the existence of an “immunological surveillance mechanism” against tumor cells. Lewis Thomas (Figure 2) suggested that the immune system recognize newly arising tumors through the expression of tumor specific neo-antigens on tumor cells and eliminate them, similarly to homograft rejection, maintaining tissue homeostasis in complex multicellular organism [2]. The first clear demonstration of specific capability to stimulate an immune response was made by Gross in 1953 after intradermal immunization of C3H mice, obtained by continuous brother to sister mating for more 20 years, against a sarcoma [3], followed by Foley in 1953 in methylcholantrene-induced tumors [4].

**Figure 3:**
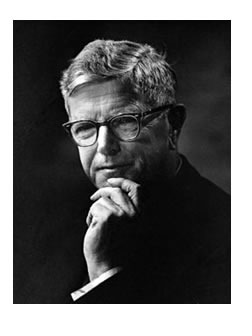
A portrait of Frank MacFarlane Burnet .

Sir Frank Mac Farlane Burnet (Figure 3) hypothesize that tumor cell neo-antigens induce an immunological reaction against cancer and subsequently formulated the immune surveillance theory [5, 6]. He wrote that: “It is by no means inconceivable that small accumulation of tumor cells may develop and because of their possession of new antigenic potentialities provoke an effective immunological reaction with regression of the tumor and no clinical hint of its existence.” [6].

## EVIDENCES CONFIRMING THE THEORY OF IMMUNE SURVEILLANCE

In transplantation models, tumors are rejected in syngeneic hosts, while transplantation of normal tissues are accepted, confirming the existence of tumor-specific antigens [6].

Professional antigen presenting cells process and present tumor associated antigens (via cross presentation of debris, perhaps due to spontaneous tumor cell lysis, or perhaps due to natural killer (NK) cell destruction, or other processes such as “nibbling”) to immune cells, and generate memory and effector cells which survey the body, seeking out tumor cells. In different types of human tumors, including melanoma, cancer of breast, bladder, colon, prostate, ovary, rectum, and glioblastoma [7–13], a longer survival has been observed in patients with an higher number of lymphocytes and NK cells . These latter do not require prior sensitization for efficient tumor cell lysis and following activation with interleukin-2 (IL-2), NK cells can kill tumor cells [15].Regulatory T cells (Tregs) exert both detrimental and beneficial effects to the host [16, 17]. Tumor antigens can be recognized by T cells, in cooperation with major histocompatibility complex (MHC) allowing T cells to interact with the antigen presenting cells [18].

## EVIDENCES AGAINST THE THEORY OF IMMUNE SURVEILLANCE

Athymic nude mice, traditionally considered to lack T cells, did not develop significantly more spontaneous or methylcholantrene-induced tumors than control mice [19, 20]. In this experimental condition, the immune response mediated by T and NK cells was similar in immunocompetent and nude mice. Interferon gamma (IFNγ) and perforin, are both involved in prevent tumor formation in mice [21, 22]. In fact, neutralization of IFNγ resulted in rapid growth of tumors [23], and mice lacking IFNγ were more sensitive to methylcholantherene-induced carcinogenesis [21]. However, treatment with IFNγ had no benefit for patients with different type of tumors [24–26]. Perforin inhibited B cell lymphoma development [27–29]. Moreover, mutations in the gene encoding perforin, have been demonstrated in lymphoma patients [30].

About 5% of individuals with primary or secondary immunodeficiences and individuals subjected to therapy to prevent transplant rejection present a heightened incidence of cancer [31]. Cancers most commonly found in immunodeficient individuals are virus-associated [32], including Epstein-Barr virus-related tumors [33, 34]. Failure of human herpes virus (HHV) immune response is one of the factors involved in the pathogenesis of Kaposi sarcoma [35]. Several other cancers, have increased incidences in persons with human immunodeficiency virus (HIV)/AIDS, including hepatocellular carcinoma, which is frequently associated with infection with the hepatitis B or C virus [36]. Merkel cell carcinoma, a rare skin cancer that occurs more frequently after organ transplantation or B-cell malignancy, conditions of suppressed or disordered immunity, has an increased incidence in HIV-infected individuals [37].

Associations between different bacteria, including Helicobacter pylori and clamyidia, and higher incidence of various tumors have been described [38, 39]. Bacteria are capable of homing to tumors when systemically administered, resulting in high levels of replication locally [40, 41]. However, the frequency of non-virally induced tumors, is not increased among transplant recipients [42].

Immune competence decreases with age, the so-called “immunosenescence”, implying that decreased immunosurveillance against cancer contribute to increased disease in the elderly [43]. Cytomegalovirus (CMV) and Epstein Barr Virus (EBV) infection are determinants of immunosenescence [44].

Immunosuppression may be not associated to an increase of tumors [45, 46]. In fact, thymectomy at birth reduced the incidence of mammary adenocarcinoma [47], and immunologic reconstitution restored the susceptibility to tumor [46]. The incidence of mammary carcinomas decreases in immunosuppressed individuals [48]. Finally, leprosy and sarcoidosis which are characterized by immunosuppression, are not associated to an increased incidence of tumors [49].

## IMMUNOEDITING, A NEW APPROACH

As Sirvastava [50] said: “The immune surveillance hypothesis is often regarded as the intellectual underpinning of cancer immunology. Although the hypothesis itself has contributed little to our attempts to treat cancer through immunological means, it has profound implications for understanding the functions of the immune system.”

**Figure 4:**
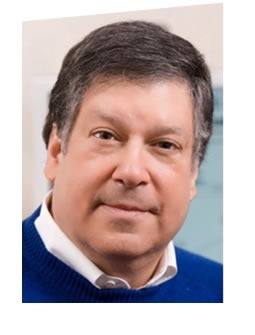
A portrait of Robert D. Schreiber

Dunn and Schreiber (Figure 4) developed the concept of “cancer immunoediting”, composed of three phases [51]. In the first one, the elimination phase, tumor cells are killed by NK, CD4+ and CD8+ cells [52]. The second phase corresponds to a state of equilibrium between immune and tumor cells. When the immune system is unable to destroy the tumor, the third phase, corresponding to the escape phase, develops which concludes with the appearance of clinically detectable tumors.

Multiple myeloma progresses from the monoclonal gammopathy of undetermined significance (MGUS) to asymptomatic and, respectively, symptomatic myeloma [53]. In this context, it is possible demonstrate that T cells from patients with MGUS develop an immune reaction to premalignant cells, which instead is absent in patients with multiple myeloma and the transition to multiple myeloma correspond to tumor escape phase [54].

The escape phase is characterized by the selection of tumor variants which will progress later on [55–57]; by a down-regulation or loss of the expression of tumor antigens; by an upregulation of resistance against tumor cells and/or an increased expression of pro-survival genes, and finally by the development of an immunouppressive tumor microenvironment [58]. Moreover, the establishment of a condition of central and peripheral immune tolerance, involving the activation of Tregs is crucial for the establishment of an escape mechanism [58, 59].

## CURRENT APPROACHES IN ANTI-TUMOR IMMUNOTHERAPY

Some analysts have predicted that within ten years, immunotherapy will constitute 60% of all cancer treatments [60]. A novel group of immunomodulatory antibodies has been introduced in the clinical use, which can break tumor specific immune tolerance and induce regression of tumors. These antibodies block growth signals of tumor cells, or induce apoptosis. Since the introduction of rituximab [61], 13 further tumor-directed antibodies have been approved.

Three of the most significant therapeutic approaches are represented by sipuleucel-T, an immunotherapeutic vaccine for prostate cancer [62]; ipilimumab, a check point inhibitor of CTLA-4 [63], and anti-programmed death receptor-1 (PD-1) and its ligand PDL-1 antibodies (anti-PD-1/PD-L-1) [64–66] for the treatment of metastatic melanoma.

Currently, cancer immunotherapies are classified as active and passive treatments. Active treatments include vaccines designed to induce tumor cell recognition. Passive treatments, on the other hand, imply direct administration of antibodies and T cells, to the patient. In this context, immune checkpoint inhibitors and adoptive T cell therapy are among the most innovative approaches [67]. At the clinical level, it is not yet clarified why certain patients respond to specific types of immunotherapies, while others do not. The development of future treatments depends on finding effective immune-based biomarkers that can help to predict responses to treatment.

## CONCLUDING REMARKS

**Figure 5:**
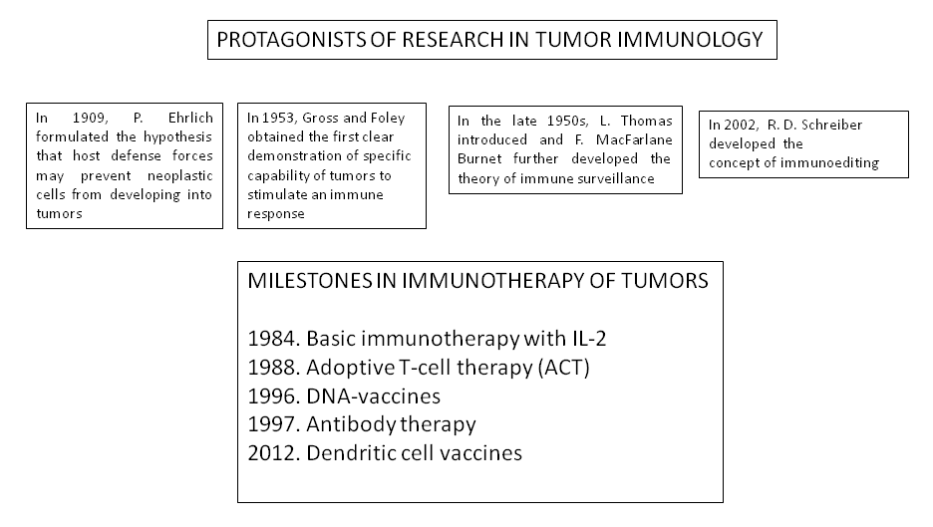
Time sheet of the protagonists of research in tumor immunology and of the milestones in immunotherapy of tumors

In this article, I have summarized the historical and experimental basis of tumor immune surveillance and cancer immunoediting (Figure 5) and I have discussed its dual roles in host protection and tumor escape. Many progresses have been found in this field starting from the original formulation of the immune surveillance theory, but further studies on cellular and molecular mechanisms to contribute to antitumor immune responses will be needed in the next years.
